# Sequencing technologies and hardware-accelerated parallel computing transform computational genomics research

**DOI:** 10.3389/fbinf.2024.1384497

**Published:** 2024-03-19

**Authors:** Michael Olbrich, Lennart Bartels, Inken Wohlers

**Affiliations:** ^1^ Center for Biotechnology, Khalifa University for Science and Technology, Abu Dhabi, United Arab Emirates; ^2^ Biomolecular Data Science in Pneumology, Research Center Borstel, Borstel, Germany; ^3^ University of Lübeck, Lübeck, Germany

**Keywords:** genome sequencing, parallel computing, computational genomics, sequencing software, pangenomics, long-read sequencing, genome references, systems genetics

## Introduction

High-throughput sequencing and hardware-accelerated computing have both developed tremendously within the last decade. In genomics, progress is fueled by new technologies that allow the sequencing of increasingly longer reads with continuously improving accuracy at steadily decreasing costs. Shifts in parallel computing arise from the observation that specific computations can be efficiently parallelized on certain hardware. Developments in the field then gained momentum when deep learning became omnipresent, which is one application that can be very efficiently parallelized on general-purpose Graphics Processing Units (GPUs).

This article discusses the impact these sequencing and hardware-accelerated computing developments have on genomic data analysis. Based on the presented observations, we provide our view on how different ongoing efforts and lines of research will come together and transform computational genomics research.

**FIGURE 1:**
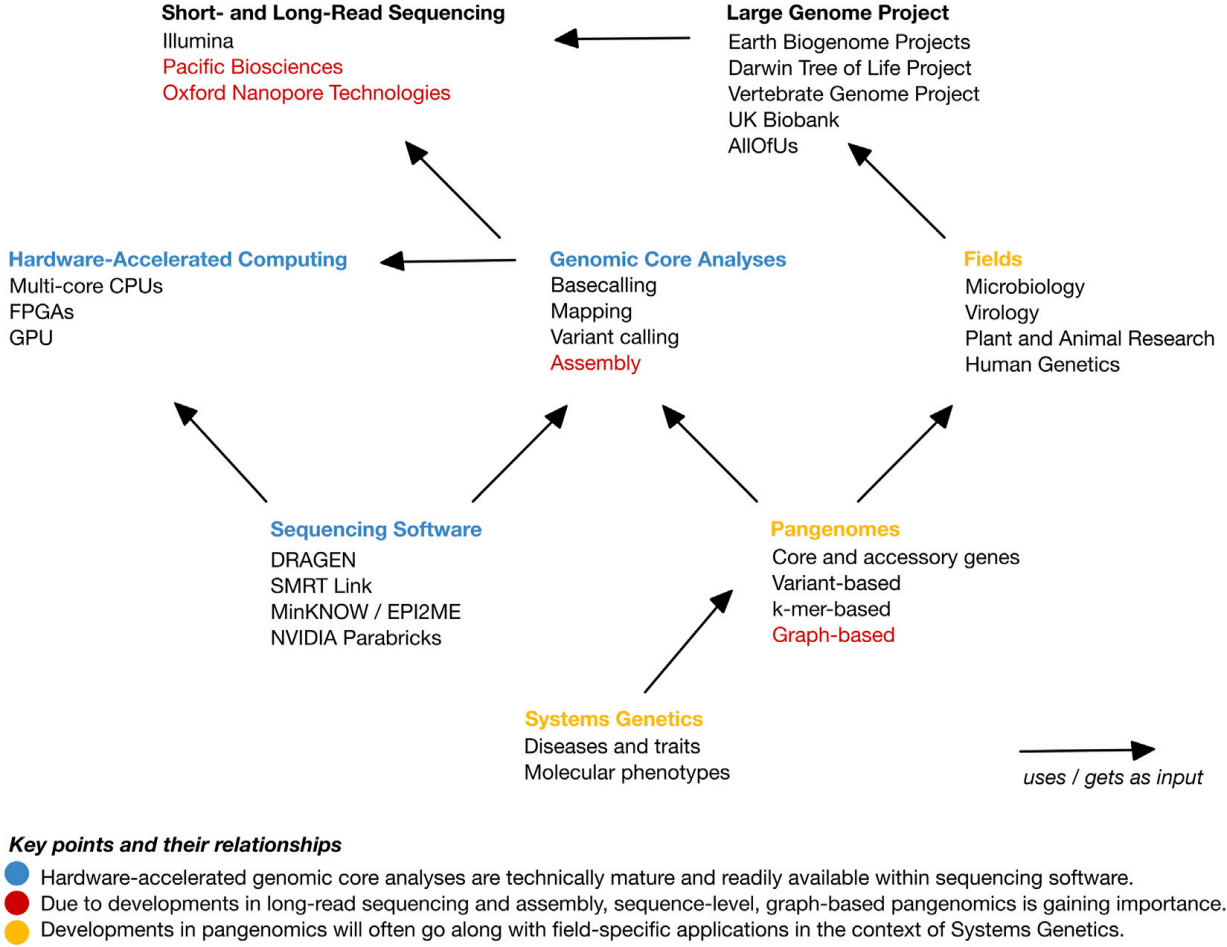
Overview of the topics of this opinion article and their relationships. The main points are highlighted in color. *Blue color:* Hardware-accelerated genomic core analyses are technically mature and readily available within sequencing software. *Red color:* Due to developments in long-read sequencing and assembly, sequence-level, graph-based pangenomics is gaining importance. *Yellow color:* Developments in pangenomics will often go along with field-specific applications in the context of Systems Genetics.

An overview of this opinion piece is depicted in Figure 1. We commence by providing a background of high-throughput sequencing and current state and endeavors in genome research. Subsequently, we briefly introduce parallel computing. We then postulate that there are four core genome sequencing data analyses: basecalling, read mapping, variant identification, and assembly. Furthermore, we elucidate the extent of their integration and the utilization of hardware-based acceleration in software suites predominantly offered by sequencing providers. Finally, we observe that currently, and across fields, a shift towards improved genomic representations, typically referred to as pangenomes, is ongoing. A pangenome represents a set of genomes or genomic sequences and is thus devised for a specific purpose. This renders approaches much more application-field specific than current general-purpose core analyses. In the Discussion, we provide our view on a forthcoming shift from representing genome data towards linking genetic data with molecular and phenotypic information, i.e., towards Systems Genetics.

## Genome sequencing developments and increase of genomic reference data

High-throughput sequencing, typically referred to as next-generation sequencing, has been around for more than two decades. Second-generation sequencing technology, dominated by Illumina, uses a sequencing-by-synthesis approach, restricting the length of reads obtained to typically 100–200 bases. Third-generation sequencing technologies have been around for the last decade, but have undergone considerable technological improvements within the last five years, resulting in gradually improved base accuracy and increased read lengths. The two different, major technologies are single-molecule real-time (SMRT) sequencing represented by Pacific Biosciences (PacBio) and nanopore sequencing, represented by Oxford Nanopore Technologies (ONT). Both technologies can generate reads with lengths up to megabases and base accuracies varying between protocols but up to more than 99%, e.g., for the PacBio high-fidelity (HiFi) protocol (Logsdon et al., 2020). These recent technological improvements made long-read sequencing a proclaimed method of the year 2022 (Marx, 2023).

Technological advancements and reduction in sequencing costs have resulted in up to a doubling of sequencing data within major sequence read archives (Arita et al., 2021) in the last five years (Katz et al., 2022), exceeding 50 petabytes (Yuan et al., 2024). Third-generation sequencing is increasingly applied, with currently more than 760,000 raw read files at the European Nucleotide Archive attributed to PacBio and Oxford Nanopore, respectively, of which about 700,000 PacBio and nearly all ONT files were submitted since 2019 (retrieved via https://www.ebi.ac.uk/ena/browser/advanced-search on 07/02/2024; search terms ‘instrument_platform = "ILLUMINA/PACBIO_SMRT/OXFORD_NANOPORE” AND first_created>2019-01-01’). In the same five-year period, about 21.3 million submitted read files were attributed to Illumina sequencing; thus, long-read sequencing files amounted to ∼7% of submissions.

Developments have also facilitated the initiation of increasingly expansive genome sequencing projects in terms of scale and scope. Examples are the Earth BioGenome Project (Lewin et al., 2022), the Darwin Tree of Life Project (Darwin Tree of Life Project Consortium, 2022), the Vertebrate Genomes Project (VGP) (Rhie et al., 2021), as well as a large number of human genome projects, among them the UK Biobank (Halldorsson et al., 2022) and AllOfUs (All of Us Research Program Investigators et al., 2019; Ramirez et al., 2022). The number of sequenced genomes also increased in plant research (Sun et al., 2022) and Microbiology (Anani et al., 2020).

## Parallel and GPU-accelerated computing speeds up computation by orders of magnitude

Parallel computing refers to utilizing multiple computing units to address a specific problem, wherein computations are executed concurrently, significantly improving computational speed. Suitable hardware resources are required on which the parallel calculations can be performed. Modern multi-core central processing units (CPUs) are capable of running many execution threads simultaneously. A special form of parallelization can be achieved by using graphics processing units (GPUs), initially designed for rapid parallel execution of mathematical calculations in computer graphics applications. With a vast quantity of processing units, GPUs surpass even the largest CPUs in terms of parallelization. GPUs have since been repurposed across various domains to accelerate tasks in which the same operation is applied to different subsets of data. Manufacturers have subsequently developed specialized GPUs and application programming interfaces to promote the development of GPU-accelerated applications. This gave rise to general-purpose computing on GPUs, the most well-known example of which is deep learning.

Acceleration by yet another order of magnitude can be achieved by concurrent computation on multiple machines. An example of such distributed computing is parallel processing on compute clusters using scientific workflow management systems such as snakemake (Köster and Rahmann, 2012) or nextflow (Di Tommaso et al., 2017), which also facilitate deployment in the cloud. Distributed computing for individual core genome analyses is a topic of interest with various tools available (Zou et al., 2021).

## Hardware-accelerated primary and secondary sequencing data analysis is technically mature and readily available

Genome sequencing data processing can be divided into primary, secondary, and tertiary analyses. Primary analysis can be considered the generation of sequence data from the sequencing devices’ raw measurements, typically in conjunction with PHRED scores that estimate individual base accuracy. This so-called base calling is typically performed during sequencing. Secondary analyses are read mapping, i.e., providing for each read the reference sequence position to which it matches, and variant calling, i.e., identifying differences from a specific reference sequence. Finally, genome assembly, i.e., reconstructing fully haplotype-phased genomes from sequencing data, will likely become an integral genomic secondary analysis of long-read data. We examined these core analyses, as well as their implementation status regarding hardware acceleration in the software solutions provided by the major sequencing companies Illumina, PacBio, and ONT.

Illumina’s DRAGEN is commercial software that can be used directly on specific Illumina machines (NovaSeq X Series, NextSeq 1000/2000), on servers on-premise, or in the cloud. It utilizes the sequencer’s onboard Field Programmable Gate Arrays (FPGAs) to accelerate primary analysis, i.e., the generation of FASTQ files from the binary base call (BCL) files. Concerning secondary analysis, DRAGEN provides the Genome Analysis Toolkit (GATK) (McKenna et al., 2010) for variant calling, which comes with a proprietary hardware acceleration that speeds up analyses significantly (Betschart et al., 2022). Besides secondary analyses, an extensive range of common tertiary analyses can be performed with DRAGEN, incurring additional licensing costs.

PacBio’s SMRT Link software includes the SMRT analysis module for secondary analysis, which provides various types of analyses of HiFi sequencing data. The variant calling workflow uses deep learning-based DeepVariant for small variant detection (Poplin et al., 2018), which can be run with GPU acceleration (Yun et al., 2021).

ONT’s MinKNOW software controls the sequencing device and offers GPU-accelerated base calling. For the secondary analysis, ONT provides the EPI2ME platform, a collection of open-source workflows that can be run free of charge.

Besides easy-to-use graphical software provided by all three major sequencing providers, major industry players, including NVIDIA, have recognized the genomic analysis market’s potential and have introduced frameworks like Parabricks that leverage GPUs to improve processing speed (Clara Parabricks 4.0.0, 2024). The acceleration of established genomic alignment and variant calling pipelines by factors of 10–100 exemplifies the impact of these technological and algorithmic developments (O’Connell et al., 2023). A notable aspect of this framework is its full accessibility to academics, with charges applied solely for commercial usage.

Deep learning is the key enabling technology for long-read base calling, with ONT using PyTorch (Paszke et al., 2019) and PacBio using TensorFlow (Developers, 2024). Accordingly, the respective latest sequencing devices are equipped with on-board GPUs. Specific deep learning achievements are improved PacBio HiFi read generation with DeepConsensus (Baid et al., 2023) and ONT models that allow detection of various base modification types (Ahsan et al., 2024) currently with basecaller Dorado.

## Pangenome approaches are increasingly applied across all research fields

The concept of pangenomes emerged in the area of microbial genomics (Tettelin et al., 2005) and has specific relevance in clinical microbiology (Anani et al., 2020). The pangenome was defined as the set of genes that occur within a bacterial phylogenetic clade, distinguishing between a core genome of shared genes, and an accessory genome of remaining genes. Besides gene-level approaches, k-mer-based approaches are used in microbiology, representing the pangenome as a presence-absence matrix of unique k-mers within contributing genomes.

Recently, pangenomics has been extended to plants (Li et al., 2022), animals (Golicz et al., 2020), and humans (Liao et al., 2023). Eukaryotic genomes are typically assessed on sequence- and not gene-level, since they contain more non-coding sequences and a larger core genome. Thus, the representation of sequence-level diversity is the primary scope of pangenomics in eukaryotes. The major benefit of sequence-level pangenomic approaches is the improvement of variant calling since variants can be identified more thoroughly with respect to a preferably diverse and comprehensive set of reference sequences.

To form a multi-genome reference, so-called pangenome graphs (Eizenga et al., 2020) are created—typically from high-quality assembled genomes. In these graphs, nodes represent subsequences and edges the adjacency of the sequences within an observed genome. Since graphs represent multiple genomes, the genetic variation within a population is captured better than with existing linear genome references (Ballouz et al., 2019; Liao et al., 2023).

Human genetics stands out as the driving force that propelled computational genomics toward harnessing the potential of long reads for high-quality assembly and subsequent pangenomic representations. This journey began with the achievement of a telomere-to-telomere assembly, encompassing all previously uncharted regions of the human genome (Miga et al., 2020; Nurk et al., 2022; Rhie et al., 2023) via diploid human assembly (Ebert et al., 2021; Porubsky et al., 2021) in conjunction with assembly algorithmic developments (e.g., Hifiasm (Cheng et al., 2021) and Verkko (Rautiainen et al., 2023)), achieving a first draft human pangenome in 2023 (Liao et al., 2023).

## Discussion

In the forthcoming decade, *de novo* assembly from long-read sequencing data stands to ascend as the coveted gold standard in many applications. Further, high-quality assembly in conjunction with pangenome representations will, for the first time, provide a complete picture of genomes and genomic variation that is not reference-biased. Such improved genomic resolution will provide opportunities to discover genotype-phenotype relationships that so far have been overlooked. This applies universally across genomes of viruses, microbes, plants, animals, and humans, all of which are currently the focus of extensive, discipline-specific genome projects. These ambitious projects, in return, catalyze the development of high-performance, hardware-accelerated implementations of largely open-source core analysis tools and corresponding graphical software for easy use with sequencing devices. In contrast, analyses that require the precise resolution afforded by long-read data, notably structural variant calling and assembly, necessitate further research and development in computational genomics. Given that these are at an earlier research stage, hardware acceleration options beyond CPU utilization have yet to be fully explored.

With the availability of longer assembled sequences up to entire genomes, pangenomic approaches will gain importance. Pangenomes, however, will often have application-specific requirements. Although pangenomic representations of genomes from high-quality assemblies are, in theory, superior to single reference-based variant lists, computational tools are still needed to construct, process, and annotate such representations for specific applications. We believe that this is a major focus of computational genomics research in the next decade and that field- and application-specific characteristics will play an important role, possibly resulting in a multitude of pangenome-centered tools. Specifically, we think that developments in pangenome representations will go along and often align with the development and application of methods that link sequence representations with phenotypes. This interplay of pangenome approaches with methods from statistical genetics and machine learning will help unlock the potential of Systems Genetics, eventually providing a holistic understanding of biological systems from the genomic viewpoint.
